# Longitudinal analysis of built environment and aerosol contamination associated with isolated COVID-19 positive individuals

**DOI:** 10.1038/s41598-022-11303-8

**Published:** 2022-05-05

**Authors:** Patrick F. Horve, Leslie G. Dietz, Garis Bowles, Georgia MacCrone, Andreas Olsen-Martinez, Dale Northcutt, Vincent Moore, Liliana Barnatan, Hooman Parhizkar, Kevin G. Van Den Wymelenberg

**Affiliations:** 1 Institute of Molecular Biology, University of Oregon, Eugene, OR 97403 USA; 2 Biology and the Built Environment Center, University of Oregon, Eugene, OR 97403 USA; 3 Energy Studies in Buildings Laboratory, University of Oregon, Eugene, OR 97403 USA; 4 Institute for Health and the Built Environment, University of Oregon, Portland, OR 97209 USA

**Keywords:** Microbiology, Environmental sciences, Engineering

## Abstract

The indoor environment is the primary location for the transmission of severe acute respiratory syndrome coronavirus 2 (SARS-CoV-2), the causative agent of coronavirus disease 2019 (COVID-19), largely driven by respiratory particle accumulation in the air and increased connectivity between the individuals occupying indoor spaces. In this study, we aimed to track a cohort of subjects as they occupied a COVID-19 isolation dormitory to better understand the impact of subject and environmental viral load over time, symptoms, and room ventilation on the detectable viral load within a single room. We find that subject samples demonstrate a decrease in overall viral load over time, symptoms significantly impact environmental viral load, and we provide the first real-world evidence for decreased aerosol SARS-CoV-2 load with increasing ventilation, both from mechanical and window sources. These results may guide environmental viral surveillance strategies and be used to better control the spread of SARS-CoV-2 within built environments and better protect those caring for individuals with COVID-19.

## Introduction

The built environment (BE)^1,2^, or the spaces that we, as humans, have built for ourselves to work in, inhabit, and enjoy life, play an essential role in mitigating the spread of severe acute respiratory syndrome coronavirus 2 (SARS-CoV-2), the causative agent of coronavirus disease 2019 (COVID-19)^3^. SARS-CoV-2 transmission indoors is almost certainly aided through extended close contact and the accumulation and persistence of aerosolized SARS-CoV-2, largely driven by poor ventilation^4–14^. Significant effort has gone into the identification of SARS-CoV-2 in a multitude of BE’s^4,6,15–28^. However, most efforts to understand the environmental contamination associated with COVID-19 individuals have been performed at a single time point, missing critical information about the longitudinal dynamics of that environmental contamination. Additionally, minimal characterization has been performed to understand how symptoms and BE factors such as ventilation, measured in air changes per hour (ACH), impact the total environmental and aerosolized contamination by SARS-CoV-2 within the BE over time.

One common scenario faced by people throughout the world is co-occupation of an indoor space with a COVID-19 positive individual while they themselves are not known to be positive. We sought to characterize the environmental viral load associated with these BE’s as they were occupied for extended periods of time. In order to better understand the longitudinal dynamics associated with the occupation of the BE when suffering from COVID-19, isolation dorm rooms housing residence hall students that tested positive for COVID-19 were sampled throughout the course of the individual’s isolation period, typically allowing for up to 10 days of sample collection. Here, we demonstrate that symptom type and symptom severity are predictive factors for the level of SARS-CoV-2 RNA environmental contamination observed and that environmental contamination decreases as individuals recover. Additionally, we provide the first real-world experimental evidence for the suppression of aerosol viral loads through the use of increased ACH from exhaust air and increased natural ventilation through the use of windows.

## Results and discussion

### Study population

A total of 35 subjects were recruited, completed an informed consent process, and participated in the study between January and May 2021. All subjects tested positive for SARS-CoV-2 RNA through shallow nasal swabs and qRT-PCR. The study cohort was made up of 17 males and 18 females between the age of 18 and 24 (Table 1). The majority of individuals in the study cohort identified as White (68.6%) followed by Hispanic/Latino/Spanish (14.3%). A full breakdown of the self-identifying ethnicity of the study cohort can be found in Table 1.

**Table 1:** Demographic data of the study subjects.

Sex at birth	Percent (n)
Male	48.6 (17)
Female	51.4 (18)

Ethnicity	Percent (n)
White	68.6 (24)
Hispanic/Latino/Spanish	14.3 (5)
Native Hawaiian/Pacific Islander	2.9 (1)
Black	2.9 (1)
Multiple	5.7 (2)
Asian	5.7 (2)

Age	Percent (n)
18–24	100 (35)

### Viral shedding and environmental contamination associate with isolation day

**Figure 1:**
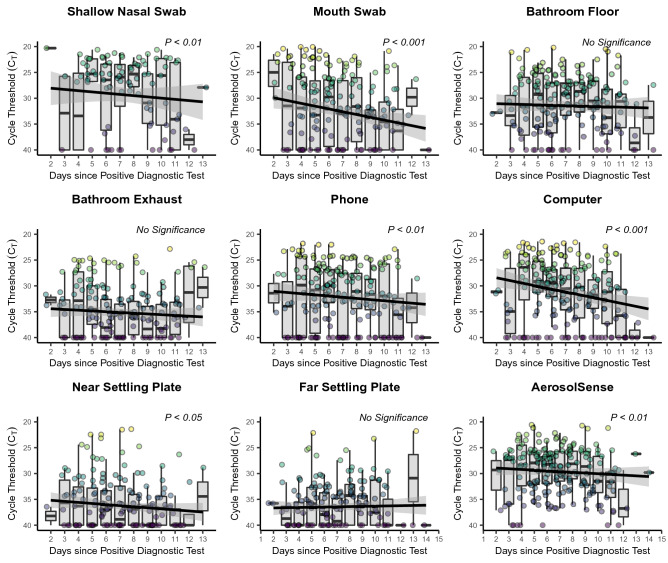
Longitudinal viral shedding and environmental contamination dynamics. The mean daily cycle threshold (C_T_) for each sampling location throughout the course of the participants’ involvement in the study. Individual points represent the mean daily C_T_ value per individual and are colored according to C_T_ value with lighter colors representing lower C_T_ values and darker colors representing higher C_T_ values. The y-axis is inverted so that lower C_T_ values are higher (to represent higher viral load) and higher C_T_ values are lower (to represent lower viral load). The black line represents a linear mixed model estimated using a restricted maximum likelihood (REML) approach and including the individual occupying the room as a random effect and the grey area represents the 95% confidence interval for that model.

**Figure 2:**
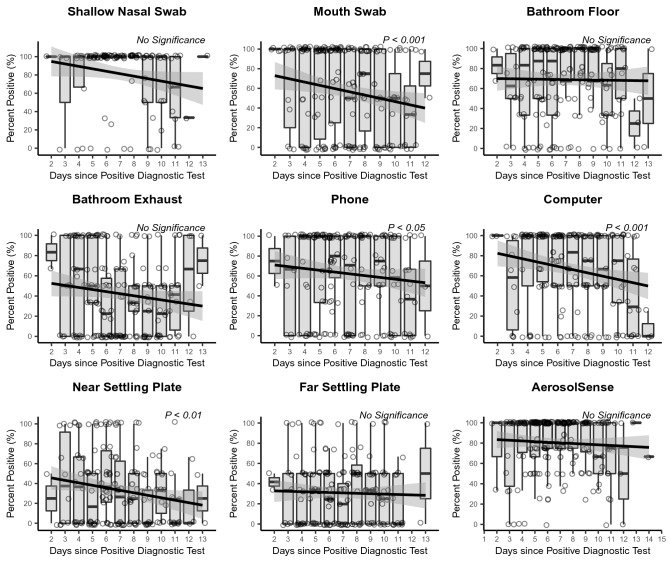
Mean daily percent positivity at each sampling location. The percent positivity rate per entry per study subject was calculated and the mean positivity rate of all participants per day enrolled in the study was calculated as the daily percentage rate. The black line represents a linear mixed model estimated using a restricted maximum likelihood (REML) approach and including the individual occupying the room as a random effect and the grey area represents the 95% confidence interval for that model.

In an attempt to assess the viral load dynamics over the course of the study cohort’s time in the isolation dormitory, the mean C_T_, a proxy for observed total viral load, of each study participant from each location was tracked throughout the course of the isolation period. C_T_ values of subject shallow nasal and mouth swabs were found to be significantly (*P* < 0.05) associated with day since positive test, with C_T_ values increasing (lower viral load) as time since positive test increases (Fig. 1). Additionally, significant increases in C_T_ values were observed as time progressed in environmental swabs taken from the study subject’s computer, phone, the settling plate closest to the study participant, and in the active air samples (AerosolSense). Statistically significant increases in the C_T_ values of participant bathroom floors, bathroom exhaust, and far passive air settling plate were not observed, although nearly all sample types trended towards increased C_T_ values over time. Furthermore, environmental samples (environmental swabs, settling plates, and active air samples) demonstrated decreasing percent positivity over time (Fig. 2).

Increasing nasal and mouth C_T_ values and decreasing rate of positivity of environmental samples as the isolation period progresses both suggest that decreasing viral load in study participants directly translates to decreased viral load within the space occupied by individuals positive for COVID-19 (Figs. 1, 2). While previous investigations have demonstrated the presence of SARS-CoV-2 RNA in BE’s occupied by COVID-19 positive individuals^4,15,20,26,28,29^, this represents the first link between infection stage, subject viral load over time, and environmental viral load. Additionally, we confirm the findings of multiple other studies that have demonstrated the persistence of SARS-CoV-2 genetic material in patient-derived samples at the end of a treatment and/or isolation period^30,31^. The persistence of environmental SARS-CoV-2 genomic material has been cited as a potential limitation in multiple sampling campaigns that utilize surface swabs to assess contamination^24,32^. The strongest trends in increasing C_T_ values among environmental samples were observed in the phone and computer swabs, and AerosolSense active air samples. In comparison to samples that did not demonstrate a significant increase in C_T_ values over time (bathroom exhaust and bathroom floor), these sampling locations were either cleaned in between sampling (phone and computer) or utilize a fresh substrate during each collection period (AerosolSense). This comparison suggests that relic RNA may compose at least a part of the RNA collected in some sampling methods and that routinely cleaned or sampling types more resistant to relic RNA collection (such as active air sampling) may provide more utility as a surveillance tool against SARS-CoV-2 than typical environmental swabbing campaigns.

### Symptom presence impacts viral shedding and environmental contamination

The presence (or lack thereof) of symptoms associated with COVID-19 positive individuals and associated viral load in patient samples (nasopharyngeal and oral swabs) has been investigated in a multitude of previous articles and significant differences have not been identified in the viral load associated with symptomatic versus asymptomatic COVID-19 infections^33–41^. Similarly, limited data regarding the relationship between symptomatic infection and environmental contamination are available and warrants continued investigation^42^. Among the symptoms that were reported by the study population, seven symptoms (coughing, watering eyes, sore throat, loss of smell, GI symptoms, congestion, and brain fog) were found to be significantly associated with altered levels of viral load in the isolation dormitory rooms (Table 2). Increased self-reported coughing, sore throat, loss of smell, and GI symptoms were associated with lower environmental C_T_ values (and thus higher viral loads), with GI symptoms and coughing most strongly correlating with decreased C_T_ values (higher viral load). In comparison, watery eyes, congestion, and brain fog were associated with increased C_T_ values (decreased viral load). Coughing while infected with COVID-19 has been estimated to produce significantly more viral particles than normal breathing^43^. This small cohort study of 35 individuals supports the hypothesis that increased respiratory expulsion from activities such as coughing would result in increased environmental contamination with SARS-CoV-2^43,44^. Furthermore, it is known, through wastewater analysis and sequencing for the surveillance of SARS-CoV-2^45^, that SARS-CoV-2 is readily emitted from and detected in stool samples in nearly half of COVID-19 positive individuals^46^. Here we observe increased viral load associated with increased GI symptoms, further supporting the potential for a fecal–oral transmission route of SARS-CoV-2 in certain circumstances. Additionally, the other symptoms associated with increased environmental viral load (sore throat and loss of smell) both implicate the upper respiratory tract. Active viral replication has been identified in the upper respiratory tract and suggests that ongoing infection and symptom onset in the upper respiratory tract may indicate increased levels of viral secretion and environmental contamination in buildings^47^.

**Table 2:** Linear correlations between the self-reported symptoms of study participants and measured cycle threshold values. Linear correlations between the self-reported symptoms of study participants and measured cycle threshold values in the environmental samples. The statistical significance of the correlation for each symptom is noted, and the slope indicates the direction of the relationship. Positive values indicate decreased environmental viral load and negative values indicate increased environmental viral load.

Symptom correlation coefficients		
Symptom	Slope	Significance level
Fever	− 0.35	Not significant
**Coughing**	− **0.52**	**< 0.001**
Sneezing	− 0.12	Not significant
Difficulty breathing	− 0.03	Not significant
Fatigue	0.13	Not significant
Headache	− 0.16	Not significant
Eyes ache	0.15	Not significant
**Eyes Watering**	**1.48**	**< 0.001**
**Sore Throat**	− **0.30**	**< 0.05**
Distorted Taste	0.06	Not significant
Loss of Taste	0.01	Not significant
Distorted Smell	0.00	Not significant
**Loss of Smell**	− **0.13**	**< 0.01**
Ears Ringing	0.37	Not significant
**GI Symptoms**	− **0.93**	**< 0.01**
**Congestion**	**1.00**	**< 0.001**
**Brain Fog**	**0.31**	**< 0.01**

Significant values are in bold.

**Figure 3:**
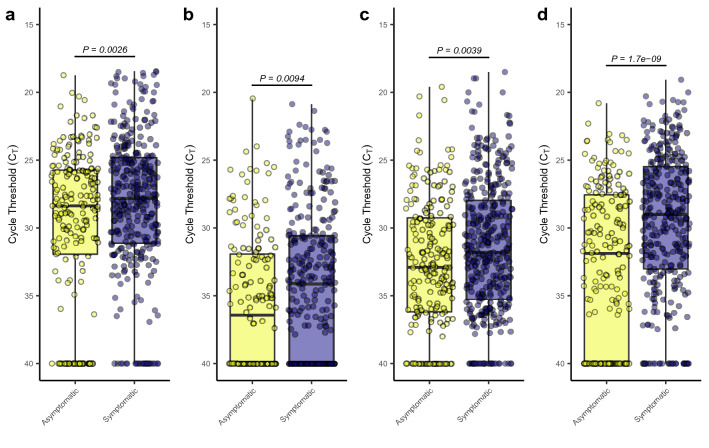
Impact of symptom presence on viral shedding and detection. (**a**) Boxplots of the observed cycle threshold values for active air samples collected by the AerosolSense sampler from rooms occupied by asymptomatic (yellow) and symptomatic (purple) individuals. (**b**) Boxplots of observed cycle threshold values for aerosol particulate samples collected by the AerosolSense sampler, passive air settling plate, and bathroom exhaust vents from rooms occupied by asymptomatic (yellow) and symptomatic (purple) individuals. (**c**) Boxplots of observed cycle threshold values for aerosol particulate samples collected by passive air settling plates and bathroom exhaust vents from rooms occupied by asymptomatic (yellow) and symptomatic (purple) individuals. (**d**) Boxplots of the observed cycle threshold values for environmental swabs collected from the computer, phone, and bathroom floor from rooms occupied by asymptomatic (yellow) and symptomatic (purple) individuals.

We sought to further understand the potential impact that symptoms play in the transmission of SARS-CoV-2 inside of the BE, and particularly, the impact symptom presence may have on subsequent environmental contamination. As such, each entry into a study participant’s room was queried to determine if the participant had self-reported any symptoms during that visit only. Individual entries were sorted into symptomatic and asymptomatic entries and the C_T_ values from each group were compared. Significantly lower C_T_ values (higher viral load; $\bar{x}=28.2$ vs. $\bar{x}=29.7$) were observed in active air samples collected during entries where the participant reported symptoms (Fig. 3a), representing greater aerosolized viral particles present during that collection time. Furthermore, significantly lower C_T_ values (higher viral load) were observed in all aerosol-based sampling methods (active air samples and passive settling plates; $\bar{x}=31.5$ vs. $\bar{x}=32.7$ and $\bar{x}=34.2$ vs. $\bar{x}=35.5$) during symptomatic entries (Fig. 3b,c). Lastly, significantly lower C_T_ values (higher viral load) were also observed in environmental swab samples collected during symptomatic visits compared to asymptomatic visits (Fig. 3d; $\bar{x}=29.6$ vs. $\bar{x}=32.7$). All together, these results suggest that the presence of symptoms, even periodically in some individuals, contributes to increased viral shedding and environmental contamination with SARS-CoV-2.

**Figure 4:**
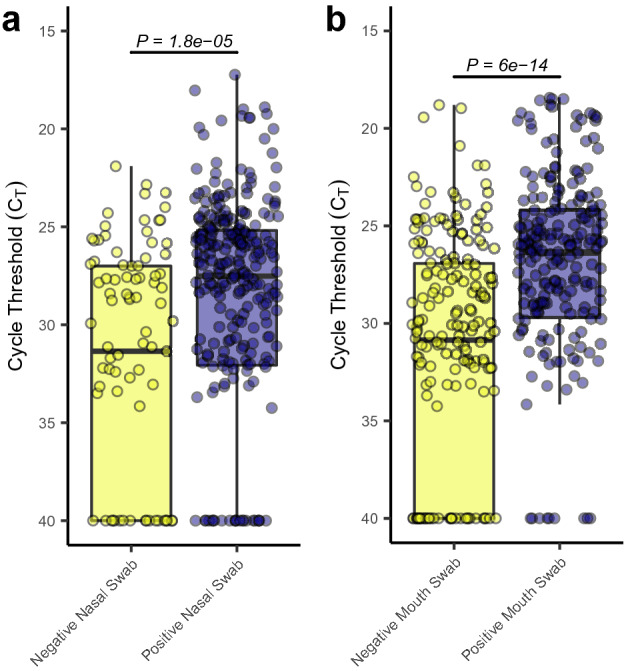
Potential intermittency of viral shedding and production. (**a**) Boxplots of the observed cycle threshold values for active air samples collected by the AerosolSense sampler from room entries when the study participant returned a negative shallow nasal swab (yellow) and a positive shallow nasal swab (purple). (**b**) Boxplots of the observed cycle threshold values for active air samples collected by the AerosolSense sampler from room entries when the study participant returned a negative oral swab (yellow) and a positive oral swab (purple).

Additionally, some subjects enrolled in the study demonstrated intermittent negative shallow nasal and oral swabs. To understand whether these periods of potentially low viral load further translated to decreased levels of aerosolized viral particles, each entry into a study participant’s room was investigated to determine whether a positive or negative swab shallow nasal and oral swabs separately) was associated with that entry. Significantly lower ($\bar{x}=29.3 \; \mathrm{vs} \; \bar{x}=32.5)$ C_T_ values (higher viral load) were observed in active air samples collected during entries where the participant returned a positive shallow nasal swab (Fig. 4a). This same statistically significant relationship $(\bar{x}=27.5\; \mathrm{vs} \;\bar{x}=31.9)$ was also observed when grouping samples based upon the result of their oral swabs (Fig. 4b). Some intermittent detection of SARS-CoV-2 RNA in the later stages of infection have been previously reported^48–50^. COVID-19 has been associated with a significant numbers of super spreader events^8,51–53^. It has been suggested that as low as 2% of COVID-19 positive individuals may account for up to 20% of confirmed cases^51^. Here, we find a potential relationship between intermittent positivity, symptom dynamics, and the detectable viral load of the subject and their environment. We hypothesize that individuals suffering from COVID-19 may undergo transient periods of viral shedding that may contribute (among many other factors) to lack of transmission in some exposure events and super spreader transmission in other exposure events.

### Built environment factors and environmental viral detectivity

**Figure 5:**
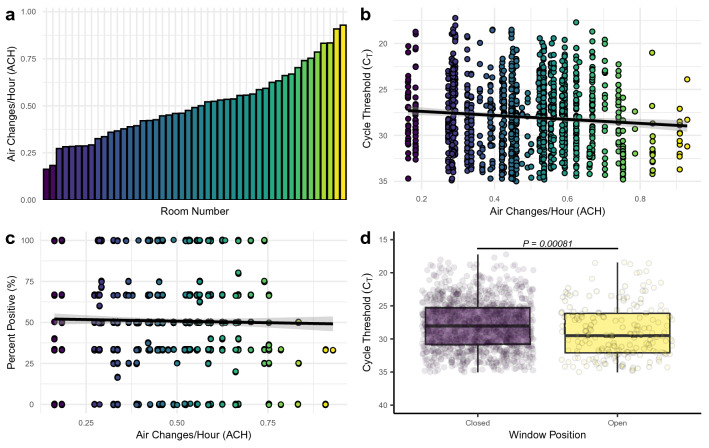
Impact of differential ventilation rates on SARS-CoV-2 RNA identification. (**a**) Distribution of the calculated air exchanges per hour (ACH) from mechanical exhaust across all isolation rooms occupied by study participants. (**b**) Relationship between the observed cycle threshold (C_T_) values and the air changes per hour (ACH) from occupied isolation rooms. The black line indicates fit from a linear model to the raw data and the grey area represents the 95% confidence interval for that model. Individual points are colored based on the ACH observed in that sample with darker colors representing lower ACH values and lighter colors representing higher ACH values. (**c**) Relationship between the observed percent positivity from each entry into a subject room and the air changes per hour (ACH) from occupied isolation rooms. The black line indicates fit from a linear model to the raw data and the grey area represents the 95% confidence interval for that model. Individual points are colored based on the ACH observed in that sample with darker colors representing lower ACH values and lighter colors representing higher ACH values. (**d**) Boxplots of observed cycle threshold (C_T_) values of aerosol samples taken during periods when the window was open for more than 50% of the sampling period (yellow) or closed for more than 50% of the sampling period (purple), as recorded during the entry surveys answered by participants.

The BE has been demonstrated to be an area of high risk when there is a COVID-19 positive individual occupying the space^5,9,10,54,55^. Despite initial guidance that SARS-CoV-2 is transmitted through droplets and close interactions between individuals^56^, it has become readily apparent that a major transmission method is through aerosolized viral particles that remain suspended in the air for extended periods of time^4,6,7,11–14,26^. As such, we sought to understand the relationship between a range of air exchange rates in the isolation rooms studied and detectability of aerosolized SARS-CoV-2. The rate of exhausted air was measured from each isolation dorm room and the air changes per hour (ACH) were calculated for each room (see “Materials and methods” for full details). The ACH from mechanically exhausted air in the isolation dorm rooms ranged from 0.16 ACH to 0.93 ACH (Fig. 5a). Current American Society of Heating, Refrigerating, and Air-Conditioning Engineers (ASHRAE) guidelines suggest a minimum of 0.35 ACH for multifamily units, 1.7 ACH for retail spaces, and 2.8 ACH for classrooms^57^. ACH from mechanical exhaust in the isolation rooms was found to be significantly and positively related to observed C_T_ values (*P* < 0.01), with increased ACH in the room more likely to produce higher C_T_ values (Fig. 5b). However, a significant decrease in the percent positivity of aerosol samples was not observed (P = 0.43) as ACH increased across study rooms (Fig. 5c). Taken together, these results suggest that, even across a fairly narrow range of ACH, increased ventilation rate decreases the detectable aerosolized viral load within enclosed spaces. However, the lack of decrease in percent positivity suggests that the modest range of ACH values found in this study is not enough to decrease the abundance of viral particles in the enclosed space to an undetectable level. Multiple articles have previously hypothesized that increased ventilation rate would translate to lower airborne viral loads^19,58–61^. To our knowledge, this study demonstrates the first real-world experimental evidence of increased ventilation within the built environment contributing to decreased aerosolized viral load.

One common method for increasing the ventilation that is available in the vast majority of BE’s is the operation of windows. Windows can dramatically increase the overall ACH within buildings and other enclosed spaces^62^. In this case, opening a dorm room window will decrease the pressure on the mechanical ventilation (the exhaust air fan in the bathroom) and increase the efficiency of air movement by the exhaust fan^63^. More importantly, the opening of a window will often increase the absolute ACH, and not just the measured ACH of the exhaust, in the room through bulk air movement in and out of the open window^64^. In order to assess the potential impact of window operations on the aerosolized viral load present within the study participant’s rooms, study participants were asked the status of their room windows during the course of the previous sampling period and researchers observed current window operation status at each entry. Samples were split into two groups consisting of (i) the window was open for more than 50% of the sampling period or (ii) the window was open for less than 50% of the sampling period. Samples from aerosol collection methods (AerosolSense and passive settling plates) demonstrated a significant increase in C_T_ values (correlating with a decrease in viral load) when the window was open for more than 50% of the sampling period (Fig. 5d). These results suggest that the increased ventilation that is provided from an open window has the ability to reduce the detectable viral load in the room by half when windows are open ($\bar{x}=34.4$) compared to when the windows are closed ($\bar{x}=33.2$). Window opening, as suggested by a variety of previous analyses and reviews^60,65–69^, appears to provide significant reduction in viral load while being a low-cost and low-labor intervention when thermal control, security, and environmental introduction is not a concern.

There are multiple limitations to note in our investigation. Our study population, made up of students living in the university residence halls, is inherently not a representative sample of the broad spectrum of individuals that may contract COVID-19. Particularly, our study population is composed of individuals between the ages of 18 and 24. The age of the individual suffering from COVID-19 has been associated with altered levels of detectable SARS-CoV-2 RNA^70^ and viral shedding dynamics may differ from that seen in our investigation. Additionally, it is important to note that the sampling locations in the rooms were static while the study participants did have the opportunity to move throughout the room. We anecdotally observed that most study participants either worked at the desk that was located in the room or remained in bed and this led to our naming of the settling plate co-located with the AerosolSense sampler as the “far sampling plate.” However, this is in fact a potentially misleading label if the study participant moved around the room throughout the course of the sampling period. Furthermore, our symptom and window position results are largely based upon the results of self-reported survey data. This survey data may suffer from inconsistencies and misclassification bias, particularly data pertaining to symptom presence and severity^71–74^. Lastly, there is a lack of data demonstrating a presence or absence of SARS-CoV-2 viability throughout the course of the study participants’ time in the isolation rooms. SARS-CoV-2 RNA has been demonstrated to remain within patient and environmental samples, even when SARS-CoV-2 viability and infectiousness has ceased^75–78^.

Overall, we present a detailed longitudinal analysis of oral, nasal, and environmental viral loads associated with individuals in a quarantine environment. We find that subject samples demonstrate a decrease, but not a ceasing, in overall viral load as their quarantine period progresses. Based upon the self-reported symptoms of study participants, we find that coughing and GI symptoms most strongly correlate with increased environmental contamination, likely through an increase in virus shedding during coughing and bowel activity and movements. Additionally, we demonstrate significant differences in environmental contamination between symptomatic and asymptomatic individuals. Lastly, we provide the first real-world experimental evidence for decreased aerosol viral load with increasing mechanical ventilation levels and demonstrate significantly reduced detectable SARS-CoV-2 in study rooms with open windows compared with those with closed windows. These results are directly applicable to those occupying common spaces with an individual known to be positive for COVID-19. We demonstrate that even asymptomatic infection with SARS-CoV-2 can yield high levels of environmental contamination. However, we also identified that increasing the total ACH within the space occupied by the COVID-19 positive individual can aid in the reduction of the overall viral load present in that environment. Furthermore, we add to the mounting evidence that SARS-CoV-2 is emitted by COVID-19 positive individuals which then disperse into the surrounding space as potentially infectious aerosols. Ideally, individuals should physically distance themselves from positive individuals, avoid shared air spaces, increase ventilation, ensure the COVID-19 positive individual wears a mask to reduce the quantity of emitted virus, and wear a mask themselves indoors.

## Materials and methods

### Institutional approval and data availability

All protocols regarding to the handling of biological materials were reviewed and approved by Advarra Institutional Biosafety Committee (IBC) (Protocol #PROTO202000132). Advarra IBC is an authorized external IBC for the University of Oregon and is registered with the National Institute of Health (NIH). All protocols relating to human subjects involved in the study were reviewed and approved by the University of Oregon Institutional Review Board (IRB) (Protocol #12292020) and all methods were performed in accordance with the relevant guidelines and regulations.

### Subject recruitment

University of Oregon COVID-19 protocols require individuals living in the residence halls to move out of their current residence and occupy an isolation dormitory room during the course of their isolation period (14 days). Individuals positive for COVID-19 were identified through the University of Oregon Monitoring and Assessment Program (MAP)^79^. Following transfer to the isolation dormitory, individuals were recruited into the program for the duration of their stay at the isolation dormitory or until they wished to be removed from the study.

### Subject questionnaire

During the first sampling period, study subjects verbally filled out a questionnaire (1st entry questionnaire) that asked participants about their infection timeline, positive test date, age, biological sex, race and ethnicity, recent travel history, lifestyle, medications taken, and symptom onset and severity. Additionally, study subjects verbally completed a followup questionnaire during each subsequent entry into the room to track their symptoms, medications taken, and the status of the study room windows. The symptoms that were tracked included fever, coughing, sneezing, difficulty breathing, fatigue, headache, aching eyes, watering eyes, sore throat, distorted taste, loss of taste, distorted smell, loss of smell, ringing ears, gastrointestinal (GI) symptoms, congestion, and brain fog. Study participants indicated whether or not they were currently experiencing any of the surveyed or other symptoms and the severity on a scale of 1–5, with 5 being the most severe. All survey answers were self-reported by the study participants.

### Airflow monitoring

The rate of air exhausted from the isolation rooms were determined for each room. The only location which is designed to exhaust air from the rooms is through the exhaust air vent located in the bathroom of each unit or an open window. The room air is supplied from either the building common areas (via a roof-top unit supplying 100% outside air) or the dormitory room windows. The velocity of exhausted air from each room was measured by placing a customized adapter with a three inch diameter outlet that rested against the exhaust air grille structural perimeter. A hot wire anemometer (TSI Incorporated, model #9565) with probe (TSI Incorporated, model #964) measured the velocity of flow at the center. The measurement was converted to volumetric flow rate using the equation $VF=\frac{0.9\times \pi \times {0.25}^{2}}{4}\times V$, where $V$ is the measured velocity at the center in feet per minute, $0.25$ is the three inch diameter outlet converted to feet, and $0.9$ is the conversion factor accounting for peak flow at the center and averaging flow across the area of the hole. The air changes per hour (ACH) flow rate was calculated using the dimensions of the study rooms as described in the architectural plans and the equation $AC{H}_{F}=\frac{VF\times 60}{v}$, where $v$ is the volume of the room in cubic feet, $60$ is the minutes in an hour, and $VF$ is the calculated volumetric flow rate. Measurements were taken with (1) the hall door, exterior window and, bathroom door closed, and (2) the hall door closed and the exterior window and bathroom door open.

### Sample collection

**Figure 6:**
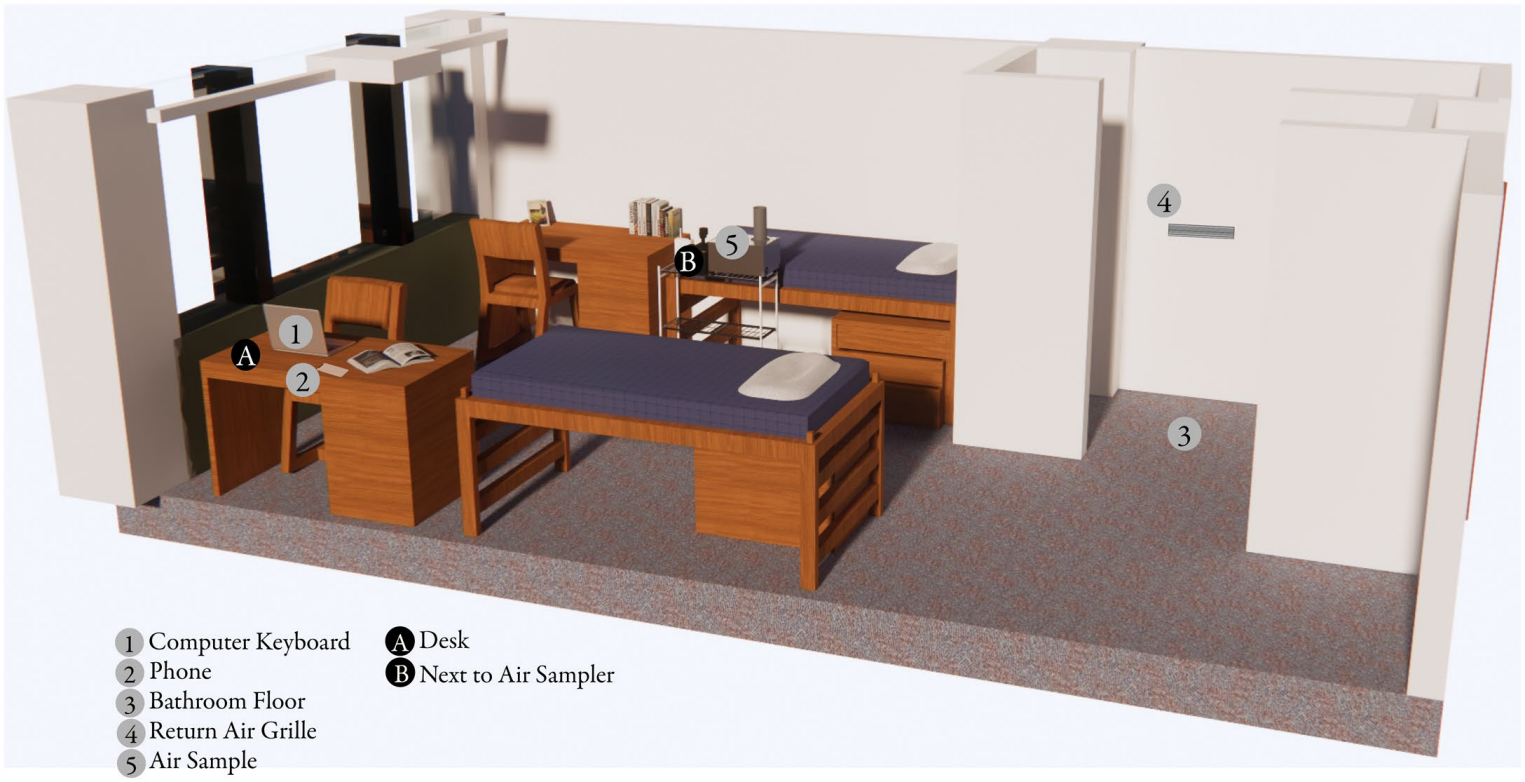
Representative layout of study rooms and sampling locations. Numbers in grey circles represent locations sampled with flocked swabs and letters in black circles represent locations sampled through passive air settling plates. Sampling location 5 represents the active air sample collected with the AerosolSense Sampler. Developed by Marin Nagle and authors using Enscape3D v.3.1 www.enscape3d.com and Adobe Illustrator v.24.2 www.adobe.com.

Samples were collected 3–5 times throughout a day with approximately 3 h (and up to 16 h for overnight collection, if participants did not turn off samplers) of sampling time occurring between subsequent sampling times. At each entry, both a mouth and shallow nasal swab were collected from the study participant. Environmental samples were collected through environmental swabs, passive air settling plates, and active air sampling (Fig. 6). Environmental swabs were collected from the participant’s cell phone, computer, bathroom floor, and exhaust air grille located within the bathroom. Flocked nylon fiber oropharyngeal swabs (Typenex Medical LLC, Catalog #SW0202) pre-moistened with DNA/RNA Shield (Zymo Research, Catalog #R1100) were used to thoroughly swab the sampling location (sampling area ~ 600 cm^2^, except for smaller items such as cellphones) for 15–20 s and returned to 1 mL of DNA/RNA Shield. Subject phones and computers were cleaned with bleach wipes following sampling to remove the residue left behind by the DNA/RNA shield. Settled particulates were captured using both components (base and lid) of standard Petri dishes (Corning Scientific). Following the sampling period, both sides of the Petri dish (sampling area ~ 110 cm^2^) were swabbed following the protocol described above for environmental swabs. Active air samples were collected using the AerosolSense 2900 sampler (Thermo Scientific, Catalog #121561-00). The AerosolSense sampler works by drawing air into an accelerating impactor at a rate of 200 L/min, causing particles to impact onto a collection substrate. Following the sampling period, the collection substrate was transferred to 1 mL of DNA/RNA Shield using sterilized forceps and transported back to the laboratory. Upon return to the laboratory, the capture media was briefly vortexed, then centrifuged for 2 min at 1500×*g* to collect all liquid from the collection substrate. Following centrifugation, the collection substrate was discarded.

### Molecular analysis

All protocols were performed in a Purifier Logic + Class II, Type A2 biosafety cabinet (LabConco, Catalog #302420001). An aliquot of 400 μL of each sample was used as the input for RNA extraction using the Quick-DNA/RNA Viral Magbead kit (Zymo Research, Catalog #R2141) following the manufacturer’s protocol. Briefly, 800 μL of lysis buffer and 20 μL magnetic beads were added to each well, the plate was sealed, and shaken continuously for 10 min. Following the ten minute incubation, the supernatant was removed, and the lysates were washed four times (1× with MagBead DNA/RNA Wash 1, 1X MagBead with DNA/RNA Wash 2, 2× with 100% ethanol). Nucleic acids were eluted into 50 μL nuclease-free water and stored at − 80 °C until downstream analysis. Successful RNA extraction was confirmed in each sample through the addition of a 5 μL spike-in of *Escherichia coli* MS2 bacteriophage into each extraction well. Each extraction plate also contained one extraction control containing nuclease-free water instead of sample.

All samples underwent quantitative reverse-transcription polymerase chain reaction (qRT-PCR) analysis using the TaqPath COVID-19 Combo Kit (Thermo Fisher Scientific, Catalog #A47814). This quadruplex qRT-PCR reaction targets the spike (S), nucleocapsid (N), and RNA-dependent RNA polymerase (RdRP/ORF1ab) genomic regions. Additionally, the assay also targets the Escherichia coli MS2 bacteriophage as an internal process control. The reaction mixtures included 5 μL TaqPath 1-Step Multiplex Mastermix without ROX (Thermo Fisher Scientific, Catalog #A28521), 9 μL nuclease-free water (Invitrogen, Catalog #4387936), 1 µL COVID-19 Real Time PCR Assay Multiplex Mix (Thermo Fisher Scientific, Catalog #A47814), and 5 µL of template RNA. Thermocycling was performed with the QuantStudio5 (Applied Biosystems) using the following cycling conditions: 25 °C for 2 min, 53 °C for 10 min, 95 °C for 2 min, and 40 cycles of 95 °C for 3 s and 60 °C for 30 s. Samples were considered positive if amplification was observed in two of three genome targets with a cycle threshold (C_T_) value less than or equal to 35 (C_T_ < 35). Each qRT-PCR plate contained a positive RNA control, a no-template control (nuclease-free water), and three extraction controls. All controls performed as expected.

### Statistical analyses

Analyses were performed using the statistical programming environment R^80^. Collected data was assessed for normality using a density plot and quantile–quantile plotting (Supplementary Fig. 1). A slight skew towards lower C_T_ values was observed, likely due to a combination of our selected study population (COVID-positive individuals) and the hard positive-sample cutoff C_T_ of 35. Associations between observed C_T_ values and study subject symptoms were identified through the use of a generalized linear model of the form $y={\beta }_{1}({x}_{1})+{\beta }_{2}({x}_{2})+...{\beta }_{n}({x}_{n}+E)$ where $y$ is the observed C_T_, ${\beta }_{i}$ values are linear regression coefficients for fixed effects ${x}_{i}$, and $E$ is a vector of errors. Significant changes in C_T_ values over time were identified through linear mixed models of the form ${y}_{i}={X}_{i}\beta +{Z}_{i}{u}_{i}+{\epsilon }_{i}$^81,82^ using a restricted maximum likelihood (REML) approach and including the individual occupying the room as a random effect. Student’s t-tests were used to compare differences in observed C_T_ values between sampling groups. Differences were considered significant with *P* < *0.05*.

## Supplementary Information

Supplementary Figure 1.

## Data Availability

All data and code supporting this study and required to recreate the analyses are deposited in Github at https://github.com/BioBE/UO-COVID-Dorms. The packages broom^83^ (version 0.7.8), dplyr^84^ (version 1.0.7), flextable^85^ (version 0.6.6), ggplot2^86^ (version 3.3.5), ggpubr^87^ (version 0.4.0), ggsignif^88^ (version 0.6.2), lmerTest^89^ (version 3.1-3), lme4^90^ (version 1.1-27.1), lubridate^91^ (version 1.7.10), plyr^92^ (version 1.8.6), scales^93^ (version 1.1.1), tidyverse^94^ (version 1.3.1), viridis^95^ (version 0.6.1), and wesanderson^96^ (version 0.3.6) were utilized in the analysis and presentation of all data.
