# Supplementary material for: Longitudinal analysis of built environment and aerosol contamination associated with isolated COVID-19 positive individuals
Source: Sci Rep. 2022 May 5;12:7395. doi: 10.1038/s41598-022-11303-8 (PMC9070971; doi:10.1038/s41598-022-11303-8)
Supplement: Supplementary file 1 — Supplementary Figure 1. [file 41598_2022_11303_MOESM1_ESM.pdf]

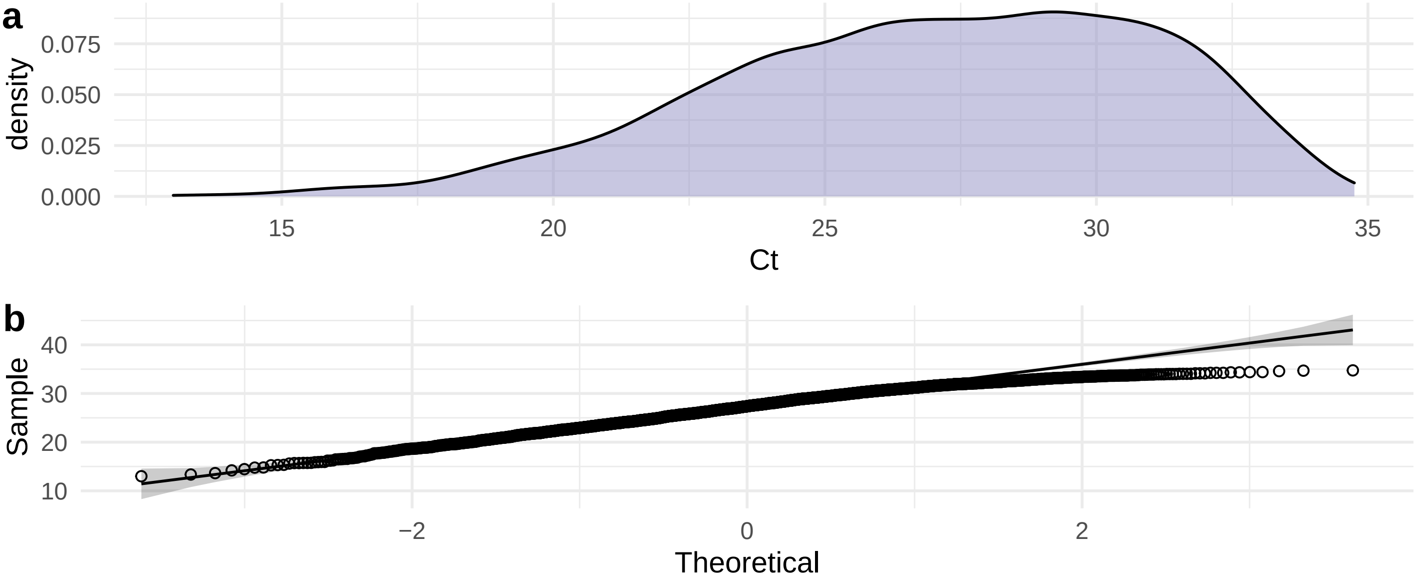

Supplmentary Figure 1. Normality of collected data. (a) Density plot of cycle threshold (Ct) values of all SARS−CoV−2 positive samples and (b) quantile−quantile plot of Ct values. Positive samples were considered positive if the Ct value was less than or equal to 35. If this was not the case, the sample returned no Ct value and was removed from the analysis. This artifact of the analysis process introduces the skewing of the normality to lower Ct values and can be observed in both the (a) density plot and the (b) quantile−quantile plot.
